# Comparative Studies on Crystallinity, Thermal and Mechanical Properties of Polyketone Grown on Plasma Treated CVD Graphene

**DOI:** 10.3390/polym13060919

**Published:** 2021-03-17

**Authors:** Sunghun Cho, Jun Seop Lee, Hyeji Jang, Seorin Park, Ji Hyun An, Jyongsik Jang

**Affiliations:** 1 School of Chemical Engineering, Yeungnam University, Gyeongsan 38541, Korea; sdfg8396@yu.ac.kr (H.J.); 21611948@yu.ac.kr (S.P.); 2 Department of Materials Science and Engineering, College of Engineering, Gachon University, Seongnam 13120, Korea; 3 School of Chemical and Biological Engineering, College of Engineering, Seoul National University, 599 Gwanangno, Gwanakgu, Seoul 08826, Korea; safe210@snu.ac.kr (J.H.A.); jsjang@plaza.snu.ac.kr (J.J.)

**Keywords:** polyketone, plasma treatment, crystallinity, CVD, graphene

## Abstract

In this work, we report a facile way to control crystalline structures of polyketone (PK) films by combining plasma surface treatment with chemical vapor deposition (CVD) technique. The crystalline structure of PKs grown on plasma-treated graphene and the resulting thermal and mechanical properties were systematically discussed. Every graphene sheet used in this work was produced by CVD method and the production of PKs having different crystallinity were performed on the O_2_- and N_2_-doped graphene sheets. It was evident that the CVD-grown graphene sheets acted as the nucleating agents for promoting the crystallization of β-form PK, while suppressing the growth of α-form PK crystals. Regardless of the increase in surface roughness of graphene, surface functionality of the CVD-grown graphene was found to be an important factor in determining the crystalline structure of PK. N_2_ plasma treatment of the CVD-grown graphene promoted growth of the β-form PK, whereas the O_2_ plasma treatment of CVD graphene led to transformation of the unoriented β-form PK into the oriented α-form PK. Thus, the resulting thermal and mechanical properties of the PKs were highly dependent on the surface functionality of the CVD graphene. The method of controlling crystalline structure of the PKs suggested in this study, is expected to be very effective in realizing the PK with good processability, heat resistance and mechanical properties.

## 1. Introduction

Aliphatic polyketone (PK) is an alternating copolymer composed of units of olefin and carbon monoxide (CO) [1,2,3,4]. PK shows incredible impact strength, abrasion resistance, chemical resistance and low gas permeability [1,2,3,4,5,6]. Production of PK also contributes to the effective consumption of CO, the representative air pollutant of the earth [1]. Therefore, the PK has great potential as a new class of environmentally friendly and high-performance engineering plastics. [5,6] The first generation PK was an alternating copolymer of an ethylene monomer and a CO monomer. The aliphatic PK with the repeating unit of ‒(CH_2_‒CH_2_‒C=O)_n_‒ has relatively high melting temperature (T_m_) between 255 and 260 °C [2]. Although the high melting point of the ethylene-carbon monoxide copolymer is useful for preventing thermal deterioration of the polymer, there is a problem of brittleness, stiffness and processability because of the high degree of crystallization of the ethylene-carbon monoxide copolymer [2,3,4]. To solve this problem, the latter generation PK could be achieved by adding a small amount of propylene monomer to the PK backbone. The latest generation of PK contains repeating units of ‒(CH_2_‒CH_2_‒C=O)_n_‒(CH_2_‒CH_2_(CH_3_)‒C=O)_m_‒ and the generated PK has lower crystallinity, lower T_m_ and better processability than the PK of the previous generation [2,3,4]. Therefore, effective control of the crystalline structure of PK has become a very important part of practical application of PK.

The crystalline forms of PKs can be classified into high-density α-phase (1.383 g cm^−3^) and less dense β-phase (1.297 g cm^−3^) [2,3]. Although both the α- and β-phases consist of orthorhombic and all-trans chain conformations, the mode of chain packing between these two crystalline structures is different. For example, the carbonyl group (C=O) is packed more densely on the α-phase than the β-phase. To obtain PK with good processability and low T_m_, it is necessary to find an effective method for promoting the formation of β-phase of PK. It is possible to increase the β-phase content of PK by increasing the propylene content during copolymerization of PK. Lagaron et al. reported that the growth of β-form crystals becomes remarkable when introducing more than 2.5 mol% of propylene inside the PK structure [4]. Furthermore, the electrospinning provides an effective mean to transform the crystalline structure of PK from α-form into β-form [3]. In the electrospinning method, solution concentration and solution viscosity are key factors to determine the resulting crystalline structures of the PK. However, the unoriented β-form PK exhibit reduced heat resistance and lower mechanical properties compared to the oriented α-form polyketones [2]. Therefore, it is required to develop a new method capable of effectively producing polyketone crystals having excellent processability while maintaining the advantages of α-form PK.

Graphene, a two-dimensional carbon nanomaterial with *sp*^2^-hybridized carbon atoms, has attracted great interest as an efficient reinforcement of polymer composites due to its high theoretical surface area (2640 m^2^ g^−1^), high Young’s modulus (0.5–1.0 TPa), tensile strength (130 GPa), chemical stability, flexibility and unique photoelectric properties [7]. Chemical vapor deposition (CVD) method is a very attractive tool for the large-scale production of graphene with more uniform thickness and less aggregation compared to the chemically converted graphenes (CCGs) [8,9,10]. In the CVD processes, gaseous hydrocarbon precursors (methane, ethane, propane) are usually decomposed on metal substrates, such as nickel (Ni) or copper (Cu) to produce graphene sheets [8,9,10]. Another important issue regarding graphene is the manipulation of its chemical structures. The manipulation can be carried out by replacing the part of carbon atoms with other elements, such as boron (B), nitrogen (N), sulfur (S), fluorine (F), oxygen (O) and so forth [9,10,11,12,13,14]. Recently, a number of studies on plasma processing have been conducted to manipulate the chemical structures of graphene sheets. The graphene sheet can be doped with either nitrogen (N) or oxygen (O) atoms through the plasma treatment, which can modify the surface properties of graphene sheets using O_2_ doping and N_2_ doping [9,10,11,12,13,14]. Thus, the CVD-grown graphene sheets treated by the O_2_- and N_2_-plasma methods have great potential to be used as efficient nucleating agents for controlling crystallization of polymers. However, the effect of the plasma-treated graphene sheet on the crystalline structure of the polymers has rarely been reported.

Herein, we present a study of the effects of plasma-treated and CVD-grown graphene sheets on the crystalline structure, thermal and mechanical properties of PKs. The CVD-grown graphene sheets served as nucleating agents for the crystal growth of PK spherulites. In addition, O or N atoms attached to the surface of graphene by plasma treatment can cause different intermolecular attraction with PKs. Therefore, the surface functionality of the CVD-grown graphene doped with the O or N was the most important determinant of the crystalline structure of PK. Consequently, changes in the crystallinity of PK were expected to affect the thermal and mechanical properties of PK. To understand the relationship between crystal growth and resulting properties of PK, polarized optical microscopy (POM), X-ray photoelectron spectroscopy (XPS), X-ray diffraction (XRD), Raman spectroscopy, differential scanning calorimetry (DSC), thermogravimetric analysis (TGA) and universal testing machine (UTM) were used.

## 2. Materials and Methods

### 2.1. Materials

Polyketone (PK), which has glass transition temperature (*T*_g_) of 10 °C, melting temperature (*T*_m_) of 220 °C and melt flow index (MFI) of 60 g/10 min, was obtained from Hyosung (Poketone, Seoul, Korea). The PK used in this work was an alternating terpolymer composed of ethylene, propylene and carbon monoxide (C=O) monomers. *m*-cresol was purchased from Tokyo Chemical Industry (Tokyo, Japan). Polymethyl methacrylate (PMMA, 4% in anisole), which acts as the transferring agent for CVD-grown graphene, was purchased from MicroChem Corp. (Westborough, MA, USA).

### 2.2. Fabrication of Plasma-Treated Graphene Sheets

The few-layer graphene was prepared by CVD method and the graphene sheet was grown on 25 mm-thick Copper (Cu) foil with CH_4_ gas as a carbon source. The Cu foil was placed in the furnace and H_2_ gas with flow rate of 8 sccm was introduced and kept at 90 mTorr for 30 min. The Cu foil was heated to 1000 °C and the elevated temperature was kept for 30 min. Next, 20 sccm of CH_4_ was introduced for 30 min at a total pressure of 560 mTorr. The Cu foil was cooled to 25 °C at a cooling rate of 35 °C/min under a H_2_ atmosphere. After the growth of graphene sheets, PMMA solution was coated on the graphene sheets using spin-coating method (Spin-1200D, Midas Systems Co., Ltd., Daejeon, Korea). The PMMA-coated graphenes were immersed into Cu etchant to detach the graphene sheets from the Cu foil. The detached graphene sheets were transferred to the glass substrate and the residual PMMA was removed by acetone. The plasma system used in this work was manufactured by Korea Vacuum Co. (Daegu, Korea) and the plasma system consists of a parallel plate with a radiofrequency of 13.57 MHz. The graphene sheets were introduced in the plasma chamber and the chamber was evacuated below 1 mTorr. Next, the O_2_ or N_2_ gas was introduced into the chamber at a rate of 30 cm^3^/min and the chamber pressure was kept at about 160 mTorr. Output power and time of the plasma treatment were 5 W and 120 s, respectively.

### 2.3. Isothermal Melt-Crystallization of PKs

To prepare PK solution, 1.0 g of PK was added to 9 g of *m*-cresol solution and the PK solution was vigorously stirred for 3 h and sonicated for 1 h. The sonochemically treated solution (*m*-cresol: PK = 90:10 by weight) was coated on plasma-treated graphene sheets using a Meyer Rod & Air Knife Coater at 10 cm/s followed by drying at 40 °C for 10 h. These processes resulted in 10 µm-thick PK films (Figure S1, see Supplementary Materials). POM images of PKs were recorded using a Nikon Lv100 microscope (Nikon, Japan) equipped with a custom-designed T-jump cell (CU-109, Live Cell Instruments, Seoul, Korea) containing two silver (Ag) heating stages for isothermal crystallization. The PKs deposited on the plasma-treated graphene sheets were maintained on a heating stage at 280 °C for 10 min and then transferred to another heating stage to provide the desired crystalline temperature. To compare the spherulite growth rates of the samples, the samples on the second heating stage were kept at different temperatures (190, 195, 200, 205, 210 and 215 °C).

### 2.4. Instrumental Analyses

Surface images of the graphene sheets were acquired with an atomic force microscope (AFM, Innova SPM, Veeco Instruments Inc., Plainview, NY, USA) and the AFM measurement was acquired at a scan rate of 2 Hz under tapping mode. X-ray photoelectron spectroscopy (XPS) traces were conducted using a K-Alpha XPS instrument (Thermo K-Alpha XPS, Thermo Fisher Scientific, Waltham, MA, USA.) equipped with a monochromatic Al *K*_α_ X-ray source of *hv* = 1.47 × 10^3^ eV. Before controlling XPS spectra, the XPS chamber was evacuated at a pressure of 5 × 10^−^^8^ mbar or lower pressure. Raman spectra of graphene sheets and PKs were measured from 1000 cm^−^^1^ to 3000 cm^−^^1^ on T6 (Horiba-Jobin Yvon Co., Tokyo, Japan) spectrometer by applying a 630 nm Ar ion laser. X-ray diffraction (XRD) analyses of PKs were carried out using a Bruker D8 DISCOVER X-ray diffractometer (Bruker, Germany). XRD patterns were recorded from 2*θ* = 10° to 50° by applying a Cu *K*_α_ X-ray source with *hv* = 1.91 × 10^4^ eV at 40 kV and 40 mA. Polarized optical microscopy (POM) images of PKs were recorded at a magnification of 100k using a Nikon Lv100 microscope (Nikon, Japan) equipped with a custom-designed T-jump cell (CU-109, Live Cell Instruments, Seoul, Korea) containing two heating stages for isothermal crystallization at 210 °C for 20 min. Differential scanning calorimetry (DSC) data of PKs were recorded using a differential scanning calorimeter (Q1000, TA instruments, Austin, TX, USA) with a heating rate of 10 °C/min and a cooling rate of 8 °C/min from 50 to 250 °C under a N_2_ atmosphere. Thermogravimetric analyses (TGA) of PKs were carried out using a Pyris 6 TGA analyzer (PerkinElmer Inc., Waltham, MA, USA) at a heating rate of 10 °C/min under air flow of 20 mL/min. Mechanical properties were measured using a universal testing machine (UTM, Instron-5543, Instron Co., Norwood, MA, USA) following the ASTM standard D638. The mechanical properties of samples were evaluated with a cross-head speed of 10 mm/min at a temperature of 24.5 °C under a relative humidity (RH) of 30%. A cross-sectional image of a PK/O_2_-G film was obtained using a field emission scanning electron microscope (FE-SEM, S-4800, HITACHI, LTD, Hitachi, Japan) at an accelerating voltage of 10 kV and at a magnification of ×2k.

## 3. Results and Discussion

**Figure 1:**
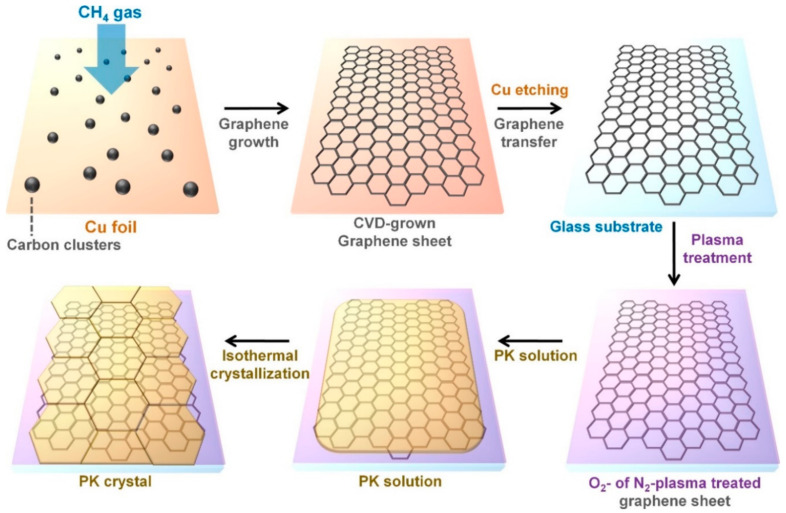
Overall procedures for crystallization of polyketones (PKs) on the plasma-treated graphene sheets.

Figure 1 demonstrates the fabrication procedures of PK films grown on the plasma-treated graphene sheets. In the CVD method, the graphene sheets were primarily grown on the copper (Cu) foil and the produced graphene sheets have been transferred onto the glass substrate. The surface of the CVD-grown graphene sheet was treated with an oxygen (O_2_) or nitrogen (N_2_) plasma and these surface-treated graphene sheets can serve as nucleating agents. Graphene sheets increase the surface roughness of substrates to grow PK spherulites, resulting in increased activation energy and reduced mobility of the PK chains [10,11,12]. Therefore, the CVD-grown graphene promotes the formation of unoriented β phase in the PK. However, O or N atoms attached to the graphene surfaces can induce different molecular interactions with the PK matrix. O_2_ plasma treatment of the graphene surface can enhance the dipole-dipole and hydrogen bond interactions between the CVD-grown graphene and the PK, resulting in higher content of the thermally stable PK crystals [9,10,11]. On the other hand, N_2_ plasma treatment of the graphene deoxygenates the graphene surface and weakens the intermolecular interaction between PK and graphene [9,12,13,14]. Because of the reduced intermolecular interaction, the N_2_ plasma-treated graphene surface causes further formation of the β-form PK. For these reasons, the plasma treatment of graphene allows selective crystallization of PK. PK terpolymer with the repeating units of ‒(CH_2_‒CH_2_‒C=O)_n_‒(CH_2_‒CH_2_(CH_3_)‒C=O)_m_‒ was chosen as a polymer matrix for interacting with the plasma-treated graphene sheets. Methylene and bisphthalasinone links of PK structure enable good solubility of PK in *m*-cresol and chloroform [15]. Thus, the preparation of the PK thin film was carried out using a *m*-cresol solvent. As-prepared PK solution was poured on the surfaces of the CVD-grown graphene sheets and then the crystallization of PK was carried out at different temperatures from 190 to 215 °C.

**Figure 2:**
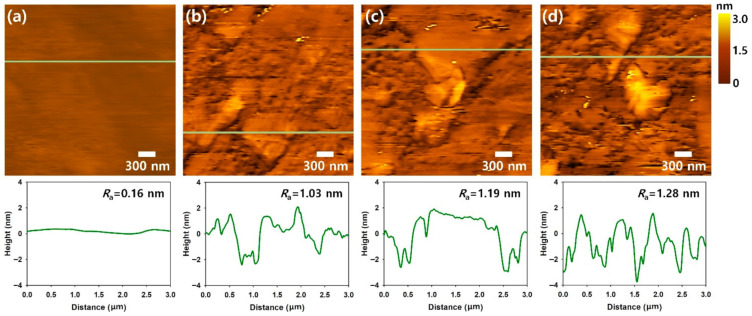
Tapping-mode AFM topographies of (**a**) Cu foil substrate, (**b**) pristine G, (**c**) O_2_-G and (**d**) N_2_-G.

To estimate the effects of N_2_ and O_2_ plasma treatments on the surface roughness of CVD-grown graphene sheets, Tapping-mode AFM of Cu foil substrate, pristine G, N_2_-G and O_2_-G are shown in Figure 2a–c. After exposing the pristine G to N_2_ and O_2_ plasma (5 W) for 120 s, slight increases in the surface roughness were observed. The surface roughness of Cu foil substrate, pristine graphene (G), N_2_ plasma-treated graphene (N_2_-G) and O_2_ plasma-treated graphene (O_2_-G) was 0.16, 1.03, 1.19 and 1.28 nm, respectively. According to previous work on the plasma-treated graphene, the graphitic carbon clusters may be conglomerated during the plasma treatments [10,11,12]. However, since the difference in surface roughness between plasma-treated graphenes and pristine graphenes was small, it is considered that the presence of graphene itself causes an increase in surface roughness.

**Figure 3:**
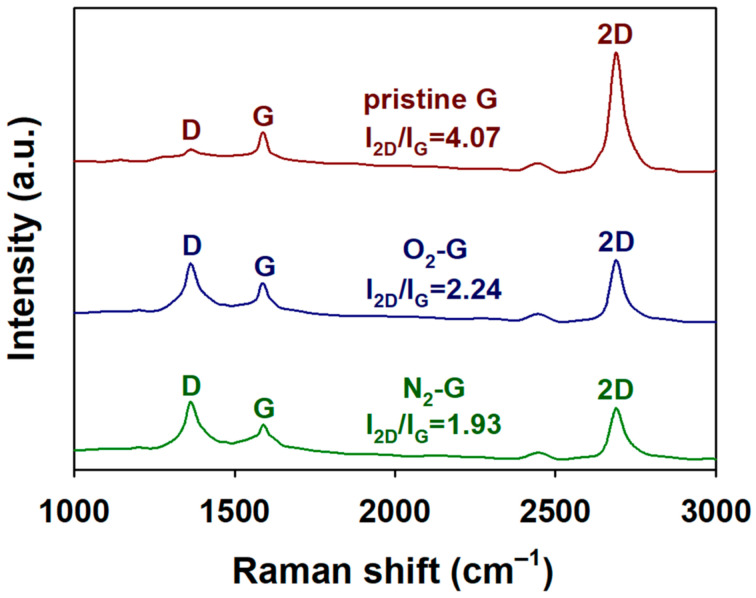
Raman spectra of pristine G, O_2_-G and N_2_-G.

Since the Raman spectrophotometer uses laser as light source, the Raman spectroscopy strengthens molecular vibrations of the graphene sheets more than the FT-IR spectroscopy. Thus, Raman spectroscopy is an effective tool for observing the structural changes in graphene sheets. Figure 3 represents the Raman spectra of pristine G, N_2_-G and O_2_-G with a laser power of 10 W for 1 min. As seen in the Raman spectrum of pristine G, the 2D peak (2688 cm^−1^) was sharper and more distinctive compared with the G peak (1591 cm^−1^) and D peak (1363 cm^−1^) and the intensity ratio of 2D to G band (I_2D_/I_G_) ratio was greater than 4. These results not only suggest that the CVD-grown graphene sheets are composed of single or double-layer sheets, but also reconfirm that the graphene sheets have a relatively low defect density [9,10,11,12,13,14,16]. The Raman spectrum of the N_2_-G showed an enhanced D peak and a reduced 2D peak compared with the pristine G. The results suggest that the nitrogen atoms embedded in the honeycomb lattice of graphene sheets destruct the structural symmetry and reduce the probability of the interatomic transitions within the graphene sheets [12,13,14]. After the O_2_ plasma treatment of the graphene sheet, an increase in the D peak and a decrease in the 2D peak were found in the Raman spectrum of O_2_-G. the intensity ratio of 2D to G band (I_2D_/I_G_) was greater than 4. This result also implies that the crystallinity of CVD-grown graphene sheets after the O_2_ plasma treatment is deteriorated [9,10,11]. In addition, the intensity ratios of D to G band (I_D_/I_G_) of pristine G, O_2_-G and N_2_-G were 0.27, 1.95 and 2.34, respectively. Considering that the I_D_/I_G_ ratio of graphene oxide (GO) sheets are generally in the range of 0.10–0.30, it can be considered O_2_-G has a completely different structure compared to GO [17,18]. Considering these results, the structural changes of the CVD-grown graphene sheets after the N_2_ and O_2_ plasma treatments are consistent with the increased surface roughness after the plasma treatment observed through the AFM.

**Figure 4:**
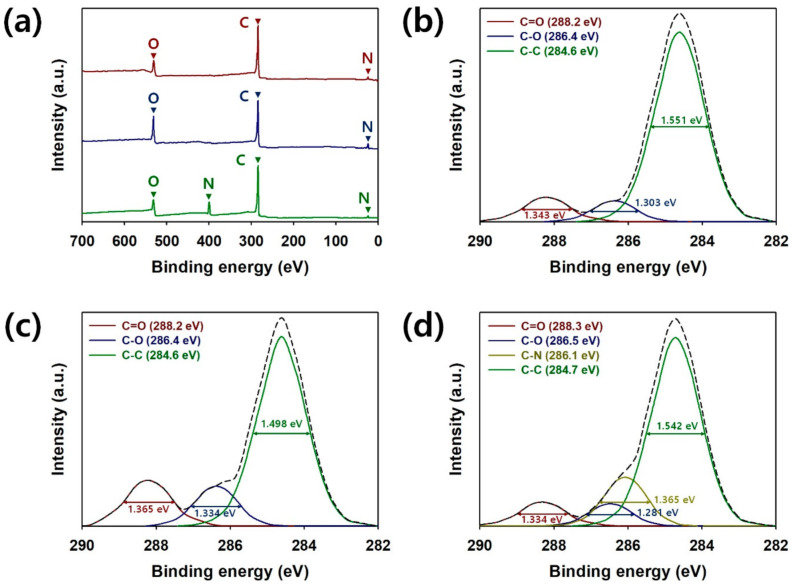
(**a**) The fully scanned X-ray photoelectron spectroscopy (XPS) spectra of pristine graphene (red), O_2_-G (blue) and N_2_-G (green). XPS core spectra in C(1s) region of (**b**) pristine G, (**c**) O_2_-G and (**d**) N_2_-G.

X-ray photoelectron spectroscopy (XPS) was used to further investigate chemical structures of the pristine G, N_2_-G and O_2_-G (Figure 4). Figure 4a represents the fully scanned XPS patterns of pristine G, N_2_-G and O_2_-G. The XPS spectra of pristine G and O_2_-G showed two peaks located at 285, 532 and 24 eV corresponding to C(1s), O(1s) and O(2s), respectively, and another peak for the N(1s) (399 eV) appeared in the spectra of N_2_-G [9,12,14,19]. Relative sensitivity factors (R.S.Fs) for C(1s), O(1s), O(2s) and N(1s) were 1.00, 2.93, 0.14 and 1.80, respectively. In the fully-scanned XPS spectrum of O_2_-G, the O content in the O_2_-G became higher compared with that of the pristine G. The atomic ratios of carbon to oxygen (C:O) for the pristine G and O_2_-G were 5.79 and 2.28, respectively. The atomic ratios of C:O and C:N for the N_2_-G were 6.58 and 4.80, respectively. These results indicate that the O_2_-plasma treatment is effective for oxygenating the surface of graphene sheets, whereas the deoxygenated surface of graphene sheets can be obtained through the N_2_-plasma treatment. Considering these results, both the O and N atoms have been successfully fixed on the surfaces of CVD-grown graphenes. Figure 4b–d represent the C(1s) spectra of pristine G, O_2_-G and N_2_-G. The peaks found at 284.6−284.7, 286.1, 286.4–286.5 and 288.2–288.3 eV were attributed to C−C, C−N, C−O and C=O bonds, respectively. Full width at half maximum (FWHM) values for C−C, C−N, C−O and C=O bonds in the C(1s) spectra were 1.498–1.551, 1.365, 1.281–1.334 and 1.334–1.365 eV, respectively. Pristine G had three major peaks at 284.6, 286.4 and 288.2 eV, corresponding to C−C, C−O and C=O, respectively (Figure 4b) [9,19]. The presences of oxygenated functional groups, such as C−O and C=O can be ascribed to the residues of PMMA or acetones. However, these C−O and C=O can be also found in the XPS spectra of pure CVD graphene shown in the previous studies. Furthermore, no characteristic peak for C=O was found in the Raman spectra of pristine G, O_2_-G and N_2_-G. Given these facts, the C−O and C=O peaks found in the spectrum of pristine G do not guarantee that the residues of PMMA or acetone still remain in the pristine G. After O_2_ plasma treatment of the CVD-grown graphene sheets, the peak intensities of C−O and C=O in the C(1s) spectrum were strengthened (Figure 4c). This indicates that more oxygenated functionalities were formed on the surfaces of O_2_-G sheets [9,19]. Peak intensities for C−O and C=O bonds were slightly weakened after the N_2_-plasma treatment, which is consistent with the fully scanned XPS pattern of N_2_-G (Figure 4d). This suggests that the N_2_ molecules can partially deoxygenate the graphene surfaces [12,14,19]. Accordingly, surface treatments of the CVD-grown graphenes have been appropriately carried out as intended.

**Figure 5:**
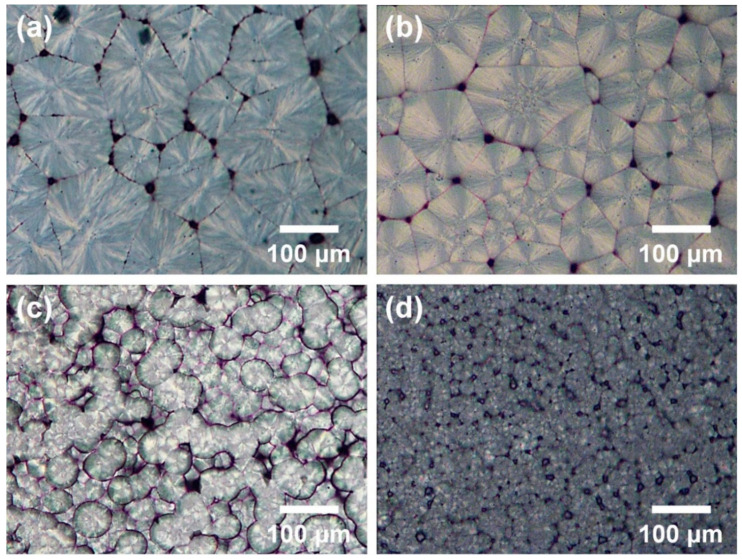
POM images of PKs fillers after isothermal crystallization at 210^o^C: (**a**) PK/O_2_-G, (**b**) pristine PK, (**c**) PK/G, (**d**) PK/N_2_-G.

**Figure 6:**
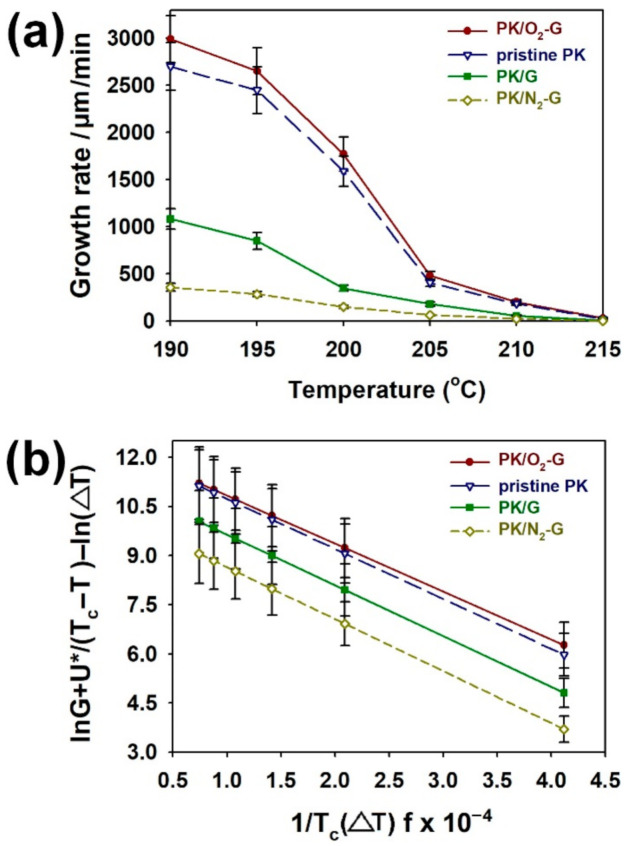
(**a**) Spherulite growth rate of PKs after isothermal crystallization at different temperatures. (**b**) Laritzen–Hoffman plots for PK/O_2_-G, pristine PK, PK/G and PK/N_2_-G.

**Figure 7:**
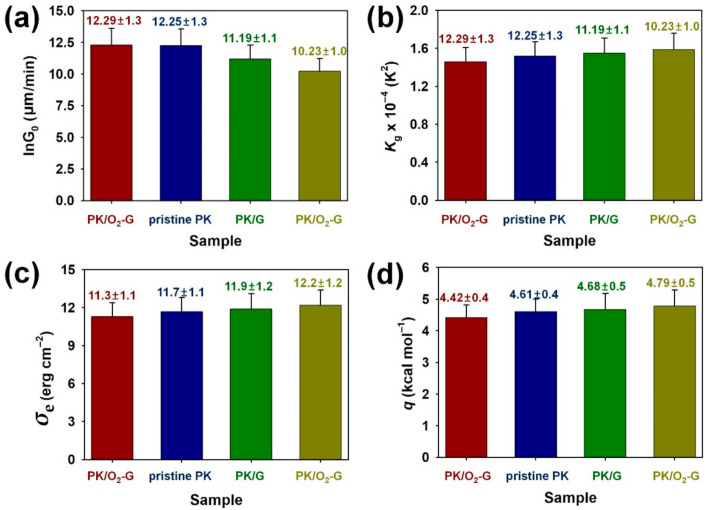
Secondary nucleation and growth parameters from spherulitic growth of PK crystals: (**a**) pre-exponential factor (lnG_0_), (**b**) secondary nucleation constant (*K*_g_), (**c**) end surface free energy (*σ*_e_) and (**d**) chain folding energy (*q*) of PK crystals.

To observe the effects of the CVD-grown graphenes on the spherulite growth of PK nanocomposites, PK nanocomposites deposited on the pristine G, O_2_-G and N_2_-G were isothermally crystallized during 20 min at 210 °C (Figure 5). After the isothermal crystallization of PKs on the surfaces of graphene sheets, the spherulite size of PK increased as follows: PK/O_2_-G > pristine PK > PK/G > PK/N_2_-G (Figure 5a–d). Moreover, same tendency was found in the spherulite growth rates of the PKs, which were estimated as functions of the crystallization temperature and time (Figure 6a). The size of the spherulite crystals grown on the graphene was smaller than the pristine PK, demonstrating that the graphene surface acts as a nucleating agent. In addition, the size of spherulites varies according to the plasma treatment method because the plasma treatment affects the free energy of the graphene surface, thereby changing the nucleating rate and chain folding energy of the PKs growing on the graphene surface [20,21]. To achieve an in-depth understanding on secondary nucleation and spherulite growth, Lauritzen-Hoffman plots {ln G + U*/[R(*T*_c_ – *T*_∞_)] – ln Δ*T versus* 1/[*T*_c_Δ*Tf*]} for PK samples are shown in Figure 6b. Δ*T* refers to the degree of supercooling (*T*_m_^0^ – *T*_c_), U* refers to the activation energy for transporting segments across the melt-crystal interface, *R* is the ideal gas constant, *T*_∞_ is the temperature below which all viscous flows stop, *f*=2*T*_c_/(*T*_m_^0^ + *T*_c_) is a function considering the temperature dependence of the heat of fusion [21]. The parameters for pristine PK are U*=1.50 kcal/mol and *T*_m_=220 ˚C [21]. The pre-exponential factor, ln *G*_0_ (the intercept), follows the order of PK/O_2_-G > pristine PK > PK/G > PK/N_2_-G (Figure 7a). This trend implies that the surface functionality either improves or reduces the adsorption of the PK chains on the graphene surface. The slope of the Lauritzen-Hoffman plot corresponds to the secondary nucleation constant, *K*_g_. The *K*_g_ is defined as an equation *K*_g_ = (*jb*_0_*σσ*_e_*T*_m_^0^)/(*k*Δ*H*_f_), where *k* is Boltzmann’s constant and *j* is 4 for crystallization regime I and III and 2 for regime II. *b*_0_ refers to the layer thickness and *σ* and *σ*_e_, refer to the lateral and end surface free energies, respectively. Δ*H*_f_ is the heat of fusion for a perfect PK crystal. The *b*_0,_ *σ* and Δ*H*_f_ values for a perfect PK crystal are 0.414 nm, 13.0 erg/cm^2^ and 227 J/g, respectively [21]. By taking these parameters into the equation of *K*_g_, the *K*_g_ × 10^4^ values for PK/O_2_-G, pristine PK, PK/G and PK/N_2_-G were 1.46 ± 0.15, 1.52 ± 0.15, 1.55 ± 0.16 and 1.59 ± 0.17, respectively (Figure 7b). Furthermore, *σ*_e_ (erg/cm^2^) values for PK/O_2_-G, pristine PK, PK/G and PK/N_2_-G were 11.3 ± 1.1, 11.7 ± 1.1, 11.9 ± 1.2 and 12.2 ± 1.2, respectively (Figure 7c). Energy of PK required for chain folding (*q*) was defined as an equation *q*=2*σ*_e_A_0_, where A_0_ refers to the cross-sectional area of the PK chain [21]. Since the A_0_ of the PK is 0.196 nm, the *q* (given in kcal/mol) values increased in the following order: PK/O_2_-G (4.42 ± 0.40) < pristine PK (4.61 ± 0.40) < PK/G (4.68 ± 0.50) < PK/N_2_-G (4.79 ± 0.50) (Figure 7d). The surface roughness of the graphene sheets acts as multiple nuclei of the PK matrix, thus increasing *K*_g_, *σ*_e_ and *q*. Despite higher surface roughness than the pristine G, O_2_ plasma treatment of the graphene surface promotes the formation of oxygenated functional groups on the graphene surface. The oxygenated functional groups on the graphene sheets consequently lower the *σ*_e_ and *q* of the PK chain [20,21]. Hence, the movement of PK chain grown on the O_2_-G will be less restrictive as the *σ*_e_ and *q* decreases, thus offering the largest spherulite size of the PK among the prepared samples [20,21]. When the graphene surface is treated with N_2_ plasma, a C-N bond is formed on the graphene sheet as described in the XPS section, which can lead to the deoxygenation of the graphene surface. Therefore, the constraint effect of N_2_-G on the spherulite growth of PK can be greater than that of the O_2_-G and pristine G [20,21]. For this reason, the N_2_-G/PK showed the largest *K*_g_, *σ*_e_ and *q* values among the PK samples. Considering from these results, graphene sheets with different surface functionalities can change the crystal growth of the PKs.

**Figure 8:**
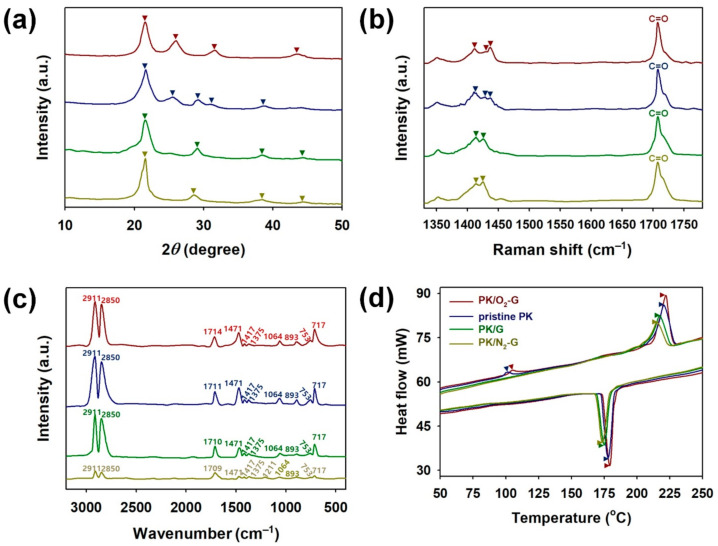
(**a**) XRD patterns, (**b**) Raman spectra, (**c**) FT-IR spectra and (**d**) DSC thermographs of PK/O_2_-G (red), pristine PK (blue), PK/G (green) and PK/N_2_-G (olive green).

To observe the effect of CVD-grown graphene sheets on the crystal structures of PKs, XRD patterns of PKs grown on pristine G, O_2_-G and N_2_-G are shown in Figure 8a. In the XRD spectra of pristine PK, several peaks appeared at 2*θ* = 21.6°, 25.9°, 29.3° and 38.7°. The peaks at 2*θ* = 21.6°, 25.9° and 31.2° correspond to the (110), (200) and (210) planes of α-form PK, respectively [2,3]. The peaks at 2*θ* = 29.3° and 38.7° are ascribed to the (210) and (310) planes of β-form PK, respectively [2,3]. These results imply that the pristine PK consists of both oriented α-PK and unoriented β-PK. When the PKs were grown on the pristine G and N_2_-G, the characteristic peaks of β-phase, corresponding to the (110), (210), (310) and (220) planes, appeared at 2*θ* = 21.6°, 29.2°, 38.6° and 44.3°, respectively, indicating that molecular defects were generated in the PK chain due to the surface roughness of the graphene sheets [2,3]. These peaks of β-phase were more prominent in the XRD pattern of the PK/N_2_-G than the PK/G, suggesting that N_2_ plasma treatment of the graphene sheet promotes the formation of β-form PK. In the XRD pattern of the PK/O_2_-G, the diffraction peaks for α-form with corresponding planes of (110), (200), (210) and (310) were observed at 2*θ* = 21.6°, 26.0°, 31.7° and 43.3°, respectively [2,3]. This indicates that the O_2_-G with lowered surface energy facilitates the movements of PK chains by enhancing intermolecular interactions with the PKs, resulting in a higher α-phase content compared to the PK/G sample. Accordingly, the O_2_-G and N_2_-G were found to be effective means for forming α-phased and β-phase crystals in the PKs, respectively. To further study the crystalline structures of PKs grown on pristine G, N_2_-G and O_2_-G, Raman spectra were investigated (Figure 8b). Several distinctive bands for PK were observed at 1412, 1428, 1438 and 1710 cm^−1^ in the Raman spectrum of the pristine PK [1,3,16]. The peaks appeared at 1412, 1428 and 1438 cm^−1^ are attributable to the α-form PK crystals [1,3,16]. The band around 1710 cm^−1^ is assigned to stretching vibration of C=O [1,3,16]. After melt crystallizations of the PKs on the pristine G and N_2_-G sheets, the peak around 1438 cm^−1^ dramatically disappeared. Furthermore, the peak at 1412 cm^−1^ shifted toward higher wavenumber, whereas the peak at 1428 cm^−1^ shifted toward shorter wavenumber. These changes are indicative of the α to β phase transition of PK [1,3,16]. In the Raman spectrum of PK/O_2_-G, the peak at 1412 cm^−1^ shifted to 1410 cm^−1^ and the peaks at 1428 and 1438 cm^−1^ shifted to 1429 and 1440 cm^−1^, respectively. The changes observed in the spectrum of the PK/O_2_-G indicate that the O_2_-G significantly enhances the formation of the α-phase in the PK chain [1,3,16]. FT-IR spectra of pristine PK, PKs grown on the pristine G, N_2_-G and O_2_-G are represented in Figure 8c. Characteristic peaks for vibration modes of PK are found at 717, 753, 893, 1064, 1375, 1417, 1471, 1709–1714, 2850 and 2911 cm^−1^ (Table 1). Peaks for symmetric and asymmetric deformation vibrations of CH_3_ were found at 1375 and 1417 cm^−1^, respectively, indicating that the PK samples used in this work includes propylene units. In particular, the peaks for C=O stretching shifted to higher wavenumber (cm^−1^) in the following order: PK/O_2_-G (1714) > pristine PK (1711) > PK/pristine G (1710) > PK/N_2_-G (1709). As the C=O groups of the PK chains can withdraw more electrons from the graphene sheets, the graphene surface weakens the intermolecular interactions between the PK chains. Therefore, the graphene sheet facilitates the formation of unoriented β-form PK crystals. Furthermore, charge transfers from N_2_ molecules immobilized on the N_2_-G sheets to PK chains are enhanced, resulting in additional blue shifts of PK/N_2_-G. The red shift of the peak for C=O in the PK/O_2_-G spectrum indicates that the O_2_-G significantly improves the intermolecular forces between the PK chains and graphene sheets, resulting in the formation of α-form PK crystals. The structural stability of the PK structure increases due to the enhancement of the intermolecular forces between the PK chains and the graphene sheets and thereby the PK chains are more compactly and densely packed. The overall peak intensities for PKs decreased in the spectra of PK/G and PK/N_2_-G, while the peak intensities for PKs found in the PK/O_2_-G became stronger. Considering these results, deoxygenated graphene surfaces contribute to the formation of α to β phase transition of PK, while the oxygenated graphene surfaces promote the formation of α-form PKs. Dynamic scanning calorimeter (DSC) thermographs of PKs grown on the pristine G, N_2_-G and O_2_-G are shown in Figure 8d. In the DSC thermograph of the pristine PK, peaks for the α to β phase transition (*T*_α‒β_) and melting (*T*_m_) were found at 101.0 and 220.5 °C, respectively. This indicates the co-existence of α and β crystal forms in the pristine PK. The exothermic peak at *T*_α‒β_ shifted to higher temperature (104.6 °C) in the thermograph of PK/O_2_-G, indicating that the O_2_-G promotes the formation of thermodynamically stable α-form PK. However, any *T*_α‒β_ peak was not observed in the thermographs of PK/G and PK/N_2_-G. This implies that the α to β phase transition is responsible for the graphene sheets [2,3]. When the PK crystals were grown on the CVD-grown graphene sheets, the *T*_m_ of PKs increased as follows (°C): PK/N_2_-G (215.2) < PK/G (217.3) < pristine PK (220.5) < PK/O_2_-G (221.8) (Table 2). In addition, crystallization temperature (*T*_c_) of PKs increased in the following order: PK/N_2_-G (173.7) < PK/G (175.1) < pristine PK (178.1) < PK/O_2_-G (179.5) (Table 2). These results indicate that the melting and crystallization behaviors of the PKs have been adjusted through O_2_ and N_2_ plasma treatment of the CVD-grown graphene sheets. The O_2_-G undergoes strong hydrogen bond interactions with the C=O group of PKs, resulting in higher *T*_m_ and *T*_c_ than the pristine PK [2,3,17,18,22,23,24]. On the other hand, the N_2_-G promote the α to β phase transition, thus lowering both *T*_m_ and *T*_c_ than both pristine PK and PK/G [2,3]. Degree of crystallinity (*X*_c_) for the PKs was calculated using equation *X*_c_ = Δ*H*_m_/Δ*H*_m_^0^ × 100% with the DSC data, where Δ*H*_m_ and Δ*H*_m_^0^ refer to the heat fusions of used sample and 100% crystalline PK (227 J g^−1^), respectively [2,3,21,22,23]. *X*_c_ was reduced after the crystallization of PKs on the N_2_-G, indicating that the deoxygenated surface of the graphene induces unoriented β phase in the PK chains. *X*_c_ of the PK/O_2_-G was about 52.5%, implying that the O_2_-G reduces the activation energy required for the chain orientation compared to the G and N_2_-G sheets (Table 2).

**Figure 9:**
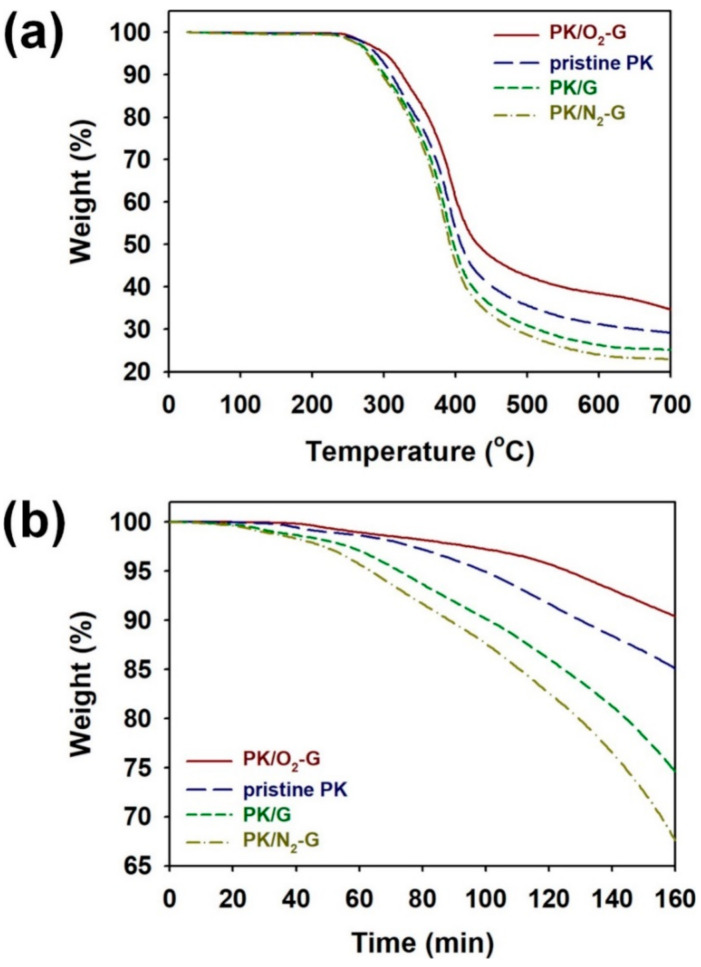
(**a**) TGA curves and (**b**) isothermal TGA curves (at 250 °C) of PK/O_2_-G, pristine PK, PK/G and PK/N_2_-G.

The thermal stabilities of PKs grown on the O_2_-G, G and N_2_-G sheets could be estimated by TGA curves shown in Figure 9. The weight losses at temperatures below 300 °C is due to C=O group in PK and the weight losses above 300 °C is ascribed to the decompositions of PK chains [17,23]. According to the TGA curves in Figure 9a, the weight losses below 300 °C for PK/O_2_-G, pristine PK, PK/G and PK/N_2_-G were 5.5, 7.7, 9.7 and 10.2%, respectively. Furthermore, the weight losses at 700 °C for PK/O_2_-G, pristine PK, PK/G and PK/N_2_-G were 65.5, 70.9, 74.9 and 78.1%, respectively. These results reconfirm that the thermal stabilities of PKs are highly dependent on the crystalline structures of the PKs. The thermal stability of the PKs was further studied by measuring the isothermal TGA curves (Figure 9b). The PKs were heated from 30 to 250 °C at a heating rate of 10 °C/min and the samples were maintained at 250 °C for 160 min under N_2_ atmosphere. After the isothermal treatment for 160 min, the residual weight (%) of PK/O_2_-G, pristine PK, PK/G and PK/N_2_-G were 90.5, 85.0, 74.7 and 68.1, respectively. Thus, the thermal stability of the PK was significantly enhanced by performing crystallization of PK on O_2_-G sheet [2,17,18].

**Figure 10:**
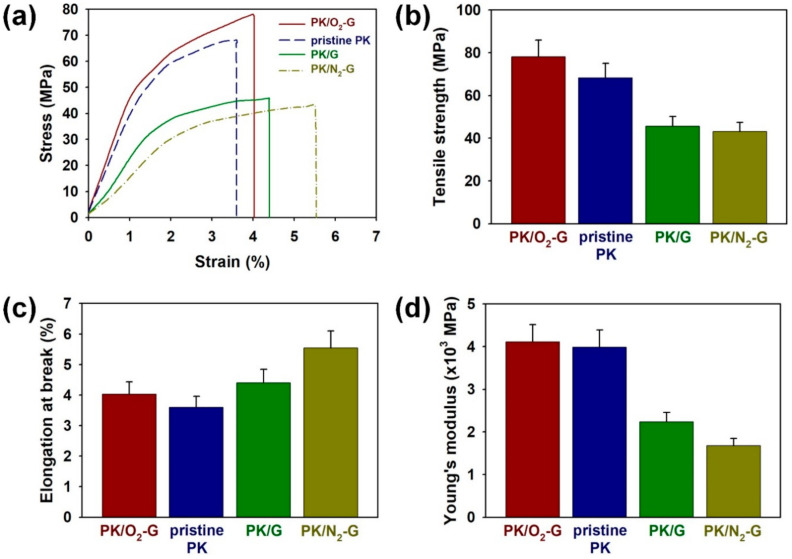
(**a**) Stress-strain curves for PK/O_2_-G, pristine PK, PK/G and PK/N_2_-G. (**b**) Tensile strength, (**c**) Elongation at break and (**d**) Young’s modulus of PK/O_2_-G, pristine PK, PK/G and PK/N_2_-G.

To evaluate the capacity of PKs to withstand tensile loads, the strain–stress (S–S) curves of pristine PK and PKs grown on different graphene sheets were recorded (Figure 10a). The tensile strength (MPa) of the PKs increased in the following order: PK/N_2_-G (43.1 ± 4.3) < PK/G (45.7 ± 4.6) < pristine PK (68.3 ± 7.0) < PK/O_2_-G (78.2 ± 7.8) (Figure 10b). As mentioned above, the O_2_-G enhances the intermolecular forces between PK chains, which improves mechanical strength of the PK [17,18,24]. The lower tensile strength of the PK/N_2_-G compared with the PK/G may be due to the higher structural defects and deoxygenated surface of the N_2_ plasma-treated graphene sheet. The elongation at break (%) of the PKs increased in the following order: pristine PK (3.6 ± 0.36) < PK/O_2_-G (4.0 ± 0.40) < PK/G (4.4 ± 0.44) < PK/N_2_-G (5.5 ± 0.55) (Figure 10c). The area under the S-S curve of the PK/O_2_-G sample was larger area than the S-S curve of the pristine PK, suggesting that the O_2_-G is effective in improving the toughness of PK [17,18,24]. The increase in the elongation at break of PK/G implies that the graphene sheets promote flexible and soft β-phase in the PKs. The phase transition from α to β was further facilitated by N_2_-G sheet, resulting in higher flexibility and toughness compared to the PK/G sample. Young’s modulus (10^3^ MPa) of pristine PK and PKs grown on O_2_-G, pristine G and N_2_-G sheets were 3.99 ± 0.40, 4.11 ± 0.41, 2.24 ± 0.22 and 1.68 ± 0.17, respectively (Figure 10d). The highest Young’s modulus of PK/O_2_-G indicates that the oxygenated surface of O_2_-G was appropriate for the formation of thermally stable and mechanically robust α-form PK. The decrease in the Young’s modulus of PK grown on N_2_-G is related to the deoxygenated surface of N_2_-G, which facilitates the formation of β-form in the PK chains [17,18,24].

## 4. Conclusions

We have studied on the crystallinity, thermal and mechanical properties of the PKs grown on the plasma-treated graphene sheets having different surface functionality. The graphene sheets used in this research were readily produced by CVD method and the CVD-grown graphene acted as a nucleating agent to promote molecular defects in the PKs. Plasma treatments were useful for successfully immobilizing O_2_ and N_2_ molecules on the surface of the graphene, resulting in different crystallinity of the PKs. PKs grown on the O_2_- and N_2_-plasma treated graphene sheets were found to consist of α-form PK and β-form PK, respectively. The O_2_-G sheet provided the effective dipole polarizations and the nucleation of α-phase crystals of the PK because of the enhanced dipole-dipole, hydrogen-bonding interactions and improved compatibility with the PK surface. The N_2_-G sheet increased the structural defects of the PK chains, resulting in the formation of β-phase crystals. In particular, these changes in the crystalline structures of the PKs directly affected their thermal and mechanical properties. The technology for controlling the crystal structures of PKs using the O_2_- and N_2_-plasma treated graphene sheets can be applied to various polymer processing technologies including extrusion molding, injection molding, roll-to-roll coating, laminating process and so forth. Consequently, this work will be the motivation to selectively produce customized PKs with different crystallinity, thermal and mechanical properties.

## Figures and Tables

**Table 1:** Secondary nucleation and growth parameters from spherulitic growth of PK crystals.

Wavenumber (cm^−1^)	Vibration
717,753	in-plane asymmetric bending (rocking) vibration of CH_2_
893	out-of-plane asymmetric (wagging) bending vibration of =C−H
1064	out-of-plane symmetric (twisting) bending vibration of =C−H
1211	stretching vibration of C−N
1375	in-plane symmetric bending (scissoring) vibration of CH_3_
1417	in-plane asymmetric bending (rocking) vibration of CH_3_
1471	in-plane symmetric bending (scissoring) vibration of CH_2_
1709–1714	stretching vibration of C=O
2850	in-plane symmetric stretching vibration of CH_2_
2911	in-plane asymmetric stretching vibration of CH_2_

**Table 2:** DSC data of PK/O_2_-G, pristine PK, PK/G and PK/N_2_-G.

Sample	T_m_ (°C)	T_c_ (°C)	T_α‒β_	∆*H*_m_ (J g^−1^)	*X*_c_ (%) ^a^
PK/O_2_-G	221.8	179.5	104.6	119.1	52.5
pristine PK	220.5	178.1	101.0	98.2	43.3
PK/G	217.3	175.1	-	74.9	33.0
PK/N_2_-G	215.2	173.7	-	61.0	26.9

^a^ The value obtained from DSC thermographs.

## Data Availability

The data presented in this study are available on request from the corresponding author.

## Supplementary Materials

The following are available online at https://www.mdpi.com/2073-4360/13/6/919/s1, Figure S1. A cross-sectional image of PK/O_2_-G sample.

## References

[1] Belov G.P., Novikova E.V. (2004). Polyketones as alternating copolymers of carbon monoxide. Russ. Chem. Rev..

[2] Klop E.A., Lommerts B.J., Veurink J., Aerts J., Van Puijenbroek R.R. (1995). Polymorphism in alternating polyketones studied by x-ray diffraction and calorimetry. J. Polym. Sci. Part B Polym. Phys..

[3] Ohsawa O., Lee K.-H., Kim B.-S., Lee S., Kim I.-S. (2010). Preparation and characterization of polyketone (PK) fibrous membrane via electrospinning. Polymer.

[4] Lagaron J.M., Powell A.K., Davidson N.S. (2000). Characterization of the Structure and Crystalline Polymorphism Present in Aliphatic Polyketones by Raman Spectroscopy. Macromolecules.

[5] You J.W., Kim J.H., Seo K.H., Huh W.S., Park J.H., Lee S.S. (2018). Implication of controlled embedment of graphite nanoplatelets assisted by mechanochemical treatment for electro-conductive polyketone composite. J. Ind. Eng. Chem..

[6] Yoon K.S., Lee J.Y., Kim T.H., Yu D.M., Seo D.W., Hong S.-K., Hong Y.T. (2014). Synthesis and properties of densely sulfonated polyketones (sPKs) with rigid backbone structure for PEM fuel cell application. J. Ind. Eng. Chem..

[7] Allen M.J., Tung V.C., Kaner R.B. (2010). Honeycomb Carbon: A Review of Graphene. Chem. Rev..

[8] You S.A., Kwon O.S., Jang J. (2012). A facile synthesis of uniform Ag nanoparticle decorated CVD-grown graphene via surface engineering. J. Mater. Chem..

[9] Iqbal M.Z., Iqbal M.W., Khan M.F., Eom J. (2015). Ultraviolet-light-driven doping modulation in chemical vapor deposition grown graphene. Phys. Chem. Chem. Phys..

[10] Mao H., Wang R., Zhong J., Zhong S., Chen W. (2014). Mildly O_2_ plasma treated CVD graphene as a promising platform for molecular sensing. Carbon.

[11] Piazza A., Giannazzo F., Buscarino G., Fisichella G., La Magna A., Roccaforte F., Cannas M., Gelardi F.M., Agnello S. (2016). Effect of air on oxygen p-doped graphene on SiO_2_. Phys. Status Solidi.

[12] Rybin M., Pereyaslavtsev A., Vasilieva T., Myasnikov V., Sokolov I., Pavlova A., Obraztsova E., Khomich A., Ralchenko V., Obraztsova E. (2016). Efficient nitrogen doping of graphene by plasma treatment. Carbon.

[13] Park S.H., Chae J., Cho M.-H., Kim J.H., Yoo K.-H., Cho S.W., Kim T.G., Kim J.W. (2014). High concentration of nitrogen doped into graphene using N_2_ plasma with an aluminum oxide buffer layer. J. Mater. Chem. C.

[14] Sakulsermsuk S., Singjai P., Chaiwong C. (2016). Influence of plasma process on the nitrogen configuration in graphene. Diam. Relat. Mater..

[15] Bazylak L.I., Zaikov G.E., Haghi A.K. (2014). Polymers and Polymeric Composites: Properties, Optimization, and Applications.

[16] Socrates G. (2004). Infrared and Raman Characteristic Group Frequencies: Tables and Charts.

[17] Lim M.-Y., Oh J., Kim H.J., Kim K.Y., Lee S.-S., Lee J.-C. (2015). Effect of antioxidant grafted graphene oxides on the mechanical and thermal properties of polyketone composites. Eur. Polym. J..

[18] Lim M.-Y., Kim H.J., Baek S.J., Kim K.Y., Lee S.-S., Lee J.-C. (2014). Improved strength and toughness of polyketone composites using extremely small amount of polyamide 6 grafted graphene oxides. Carbon.

[19] Moulder J.F., Chastain J. (1992). Handbook of X-ray Photoelectron Spectroscopy: A Reference Book of Standard Spectra for Identification and Interpretation of XPS Data.

[20] Stadlbauer M., Eder G., Janeschitz-Kriegl H. (2001). Crystallization kinetics of two aliphatic polyketones. Polymer.

[21] Holt G.A., Spruiell J.E. (2002). Melting and crystallization behavior of aliphatic polyketones. J. Appl. Polym. Sci..

[22] Miller H.A., Moneti S., Vizza F., Passaglia E., Bianchini C., Bronco S., Ceriegi S., Sulcis R., Frediani M., Ciardelli F. (2006). Polyketone Nanocomposites by Palladium-Catalyzed Ethylene-Carbon Monoxide-(Propene) Co(Ter)polymerization Inside an Unmodified Layered Silicate. e-Polymers.

[23] Marklund E., Gedde U.W., Hedenqvist M.S., Wiberg G. (2001). Properties of polyketone/polypropylene blends. Polymer.

[24] Cho J., Jeon I., Kim S.Y., Lim S., Jho J.Y. (2017). Improving Dispersion and Barrier Properties of Polyketone/Graphene Nanoplatelet Composites via Noncovalent Functionalization Using Aminopyrene. Acs Appl. Mater. Interfaces.

