# Supplementary material for: Comparative Studies on Crystallinity, Thermal and Mechanical Properties of Polyketone Grown on Plasma Treated CVD Graphene
Source: Polymers (Basel). 2021 Mar 17;13(6):919. doi: 10.3390/polym13060919 (PMC8002582; doi:10.3390/polym13060919)
Supplement: Supplementary file 1 [file polymers-13-00919-s001.pdf]

Supplementary Materials for:

Comparative Studies on Crystallinity, Thermal, and Mechanical Properties of Polyketone grown on Plasma Treated CVD Graphene

Sunghun Cho [1,*], Jun Seop Lee [2,*], Hyeji Jang [1], Seorin Park [1], Ji Hyun An [3], and Jyongsik Jang [3]

[1] School of Chemical Engineering, Yeungnam University, Gyeongsan 38541, Republic of Korea.

[2] Department of Materials Science and Engineering, College of Engineering, Gachon University, Seongnam 13120, Republic of Korea.

[3] School of Chemical and Biological Engineering, College of Engineering, Seoul National University, 599 Gwanangno, Gwanakgu, Seoul 08826, Republic of Korea.

*E-mail: shcho83@ynu.ac.kr (S.C.); junseop@gachon.ac.kr (J.S.L.)
Tel.: +82-53-810-2535

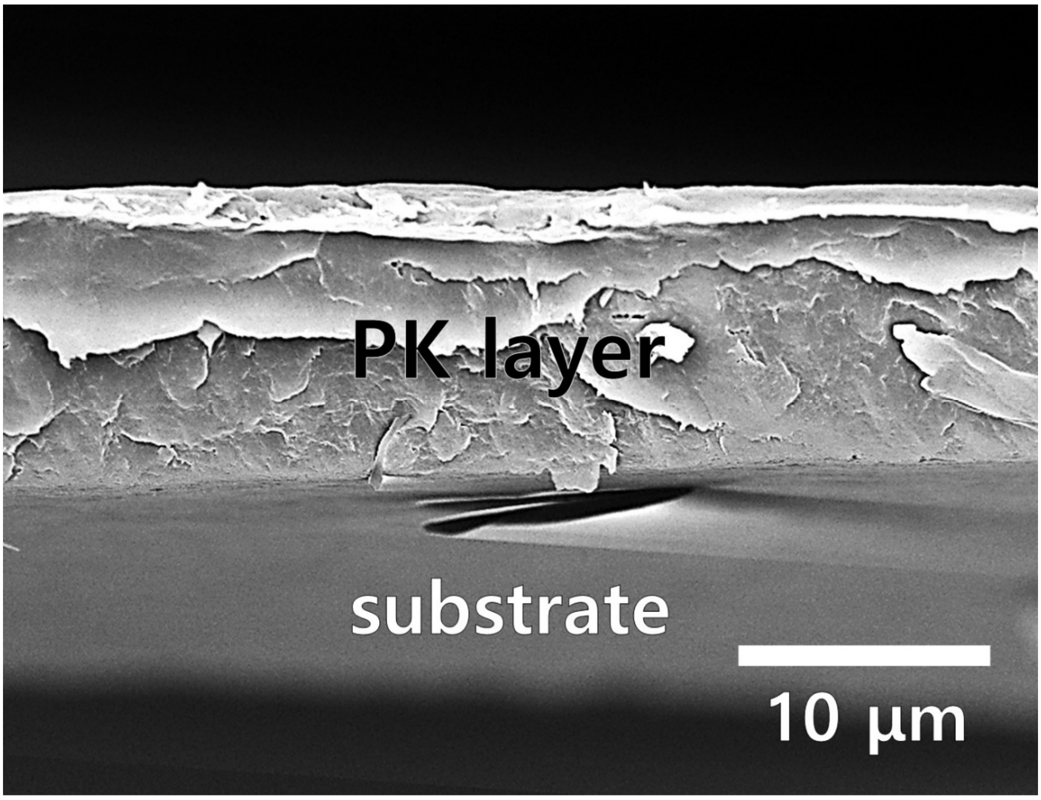

Figure S1. A cross-sectional image of PK/O_2-G sample.
